# The differences in hypoglycemic activity of Coptis herb pairs fermented by *Eurotium cristatum* and its regulatory effect on gut microbiota in T2DM rats

**DOI:** 10.3389/fmicb.2026.1753800

**Published:** 2026-03-18

**Authors:** Yanli Wang, Yincui Chen, Anqin Zhu, Jin Zhang, Shiping Lu, Chuanbo Zhang

**Affiliations:** 1 School of Life Sciences, Guizhou Normal University, Guiyang, China; 2 Department of Agronomic Engineering, Guizhou Vocational College of Agriculture, Guizhou, China; 3 Huangping Yedonghe Yuan Ecological Recycling Farming Co., Ltd., Huangping, China

**Keywords:** Coptis herbal pair, *Eurotium cristatum*, fermentation, gut microbiota, hypoglycemic effect

## Abstract

Coptidis Rhizoma-Scutellariae Radix, Coptidis Rhizoma-Glycyrrhizae Radix et Rhizoma, and Coptidis Rhizoma-Panax notoginseng are herbal pairs widely used in clinical therapy for Type 2 Diabetes Mellitus (T2DM) and gastrointestinal diseases. The fungus *Eurotium cristatum*, known for its robust enzyme system and diverse active metabolites, possesses a strong potential for the biotransformation of active constituents in Coptis-containing herbal pairs. However, the hypoglycemic effects and underlying mechanisms of these fermented pairs remain unclear. This study systematically evaluated the effects of three *E. cristatum*-fermented Coptis herbal pairs on fasting blood glucose (FBG), glucose tolerance, lipid metabolism (TC, TG, HDL-C and LDL-C), pancreatic injury protection, and gut microbiota composition in T2DM rats. The fermented Coptis-Glycyrrhiza (FRG) and Coptis-Scutellaria (FRS) demonstrated pronounced effects in improving physical condition, attenuating weight loss, and achieving stable glycemic control. Three fermented pairs regulated total cholesterol (TC) more effectively than metformin, and FRS exhibited superior protection against pancreatic islet injury. Different fermented Coptidis herb-pairs exert distinct effects on gut microbiota composition in T2DM rats. The FRS significantly enriched beneficial genera, including unclassified_f_*Oscillospiraceae*, norank_o_*Clostridia*_UCG-014, *Monoglobus* and *Ruminococcus*. The FRP significantly increased the abundance of beneficial genera such as *Lachnospiraceae*_NK4A136_group, *Monoglobus*, *Ruminococcus*, norank_o_*Clostridia*_UCG-014 and *Acutalibacter*, etc. The FRG was characterized by higher abundances of *Ligilactobacillus*, norank_f_*Lachnospiraceae*, *Akkermansia*, and *Prevotellaceae*_UCG-001. These findings suggest that the differential hypoglycemic effects of three fermented Coptis pairs are closely linked to regulation of gut microbiota dysbiosis. Our data highlight the potential of optimizing Coptis-based formulations for T2DM treatment and open new avenues for traditional Chinese medicine.

## Introduction

Coptidis Rhizoma (*Coptis chinensis* Franch), widely used for clearing heat, drying dampness, reducing and detoxifying properties, is a prevalent medicinal herb in classical formulas such as Sanhuang Xiexin Decoction, Gegen Qinlian Decoction, and Huanglian Wendan Decoction etc. Many renowned practitioners of traditional Chinese medicine in history frequently utilized Coptidis Rhizoma as an adjunctive therapy for diarrhea, inflammation, gastrointestinal infections, Type 2 Diabetes Mellitus (T2DM) and its complications. Berberine (BBR), the most representative isoquinoline alkaloid in Coptidis Rhizoma, accounting for 5% ~ 8% of its content, stimulates gut symbiotic probiotics to produce short-chain fatty acids (SCFAs), enhances the expression of insulin receptors (InsR) via the phosphatidylinositol 3-kinase (PI3K) signaling pathway, and promotes the secretion of glucagon-like peptide-1 (GLP-1), exerting hypoglycemic effects (Chen et al., 2023; Araj-Khodaei et al., 2024). Additionally, berberine inhibits SIRT3 to modulate mitochondrial metabolic pathways, promotes glucose uptake, and induces the degradation of the gluconeogenic enzyme PEPCK1 (Fang et al., 2022). However, its clinical efficacy is severely hampered by extremely low water solubility and poor oral bioavailability. When treating diabetes and its complications with Coptidis Rhizoma, the typical dosage ranges from 15 to 30 g. However, in cases of diabetic ketoacidosis, the required dosage may increase up to 120 g to achieve satisfactory blood glucose-controlling effects. Microbial fermentation of traditional Chinese medicine (TCM) formulas can efficiently metabolize and transform the active constituents of herbs, significantly improve oral bioavailability, and enhance therapeutic efficacy. This process is also promising for expanding their pharmacological activities and uncovering novel applications (Chen et al., 2022).

Traditional Chinese Medicine (TCM) emphasizes herb preparation according to established methods, herb combination based on synergistic interactions, and treatment rooted in holistic differentiation of signs and symptoms. Due to its bitter taste and cold properties, Coptidis Rhizoma was commonly paired with other herbs, notably Coptidis Rhizoma-Scutellariae Radix, Coptidis Rhizoma-Glycyrrhizae Radix et Rhizoma, and Coptidis Rhizoma-Panax notoginseng. The Coptis-Scutellaria herb pair is a widely used combination, known for its potent heat-clearing and detoxifying effects, demonstrates superior efficacy in alleviating glucose and lipid metabolism disorders compared to either herb alone, owing to its regulation of gut microbial metabolites and the MAPK/PI3K/Akt signaling pathway (Cui et al., 2018). When decocted, the herb pair produce a significant amount of flocculent precipitate, presumably, resulted from the formation of relatively weak chemical bonds, such as hydrogen bonds and intermolecular forces, between berberine and baicalin. These interactions enhance the dissolution of active constituents and facilitate the creation of a supramolecular system (Shen et al., 2024). The Coptidis-Glycyrrhiza herb pair is a classic combination, whereby Glycyrrhiza is used to counteract the bitter-cold properties of Coptidis. When decocted, the herbal combination demonstrates significant clinical benefits and enhanced heat-clearing and detoxifying effects. The underlying mechanism may involve glycyrrhizic acid facilitating the transport of berberine across intestinal epithelial cells, thereby increasing berberine plasma concentration and bioavailability, which slows its metabolism and prolongs its therapeutic efficacy (Zhang et al., 2022). *Panax notoginseng* posses the effects of activating blood circulation and unblocking collaterals, widely used to treat cardiovascular diseases. The Coptis-Notoginseng herb pair clears heat, moistens dryness, promotes blood circulation, commonly was used to treat “Xiaoke Syndrome,” and can therefore be employed in treatment of T2DM and its complications.

Probiotics exhibit great potential in maintaining gut microbiota homeostasis and treating T2DM. Particular attention has been paid to strains with excellent probiotic properties, robust enzyme systems, and strong biotransformation capabilities for the active constituents of TCM. *Eurotium cristatum*, a dominant fungus in Fu brick tea, secretes a wide array of extracellular enzymes, such as cellulase, hemicellulase, acid protease, glucose oxidase, and β-glucosidase, that efficiently break down cellulose and hemicellulose, thereby promoting the dissolution of active pharmaceutical components from TCM (Tan et al., 2024). After fermentation of white ginseng (*Panax ginseng* C. A. Meyer) with *E. cristatum*, the total flavonoid content, ferric reducing antioxidant power, scavenging activities against DPPH and ABTS radicals, as well as *α*-amylase and α-glucosidase inhibitory activities were considerably enhanced. Additionally, *E. cristatum* exhibited stronger biotransformation activity on white ginseng than *Lactiplantibacillus plantarum*, *Bacillus licheniformis*, and *Saccharomyces cerevisiae* (Chen et al., 2022). Moreover, eurocristatine, a diketopiperazine dimer alkaloid derived from *E. cristatum* and potentially inducible using TCM substrates, has been shown to improve insulin resistance in diabetic mice by activating the PI3K/AKT signaling pathway (Zhang et al., 2020). Therefore, *E. cristatum* is considered one of the best probiotic strains for fermenting TCM and biotransforming major pharmacological components.

The active components of traditional Chinese medicine (TCM), obtained through probiotic-mediated biotransformation, often exhibit enhanced pharmacological effects compared to their original precursors. For instance, a novel metabolite-oxyberberine (OBB), generated from berberine in Coptidis Rhizoma through oxidation by the gut microbiota, exhibits high bioavailability, reverses gut microbiota dysbiosis, and demonstrates superior hypoglycemic activity via regulation of pancreatic PI3K/Akt and Nrf2 signaling pathways (Li et al., 2023). Baicalin, the main active component in Scutellariae Radix, can be hydrolyzed by β-glucuronidase from probiotic microbes into baicalein, which possesses stronger anti-inflammatory, antioxidant, and preventive as well as improving effects on lipid metabolism disorders (Liu et al., 2020, 2024; Ji et al., 2024). The resulting products from fermentation of herbs with probiotic consortia, including induced active metabolites, biotransformed components, and microbial cell proteins, collectively exert a natural prebiotic effect. This effect suppresses the proliferation of harmful microorganisms while selectively promoting the colonization of indigenous beneficial microbes, thereby enabling them to re-establish as dominant taxa and maintain gut microbiota homeostasis.

Microbial fermentation offers a promising new avenue for applying TCM to T2DM treatment. However, the fermentation of different herb pairs with various microbial strains leads to the formation of fermentation products with distinct active components and pharmacological activities, potentially resulting in variations in their hypoglycemic capacity and underlying mechanisms. To compare the hypoglycemic activity and underlying mechanisms among *E. cristatum*-fermented Coptis-Scutellaria, Coptis-Glycyrrhiza, and Coptis-Notoginseng herb pairs, We systematically evaluated the effects of these fermented herb pairs on blood glucose, blood lipid levels, pancreatic tissue protection, and the diversity and abundance of gut microbiota in the T2DM rats.

## Materials and methods

### Experimental animals and materials

Four week old male Sprague–Dawley (SD) rats [specific pathogen-free (SPF) grade], weighing 110 ~ 140 g, were purchased from Beijing Huafukang Bioscience Co., Ltd. (License No.: SCXK (Jing) 2019–0008). The high-fat high-sucrose diet (HFSD) consisted of 59% basal diet (72% corn, 10% wheat, 15% soybean meal, 3% premix), 20% sucrose, 10% lard, 10% egg yolk powder, and 1% cholesterol. Coptidis Rhizoma (CR), known as Huang Lian in Chinese, is the dried rhizome of several medicinal plants from the family Ranunculaceae, including *Coptis chinensis* Franch., *Coptis deltoidea* C. Y. Cheng et Hsiao, and *Coptis teeta* Wall. Scutellariae Radix (SR), commonly known as Huang Qin, refers to the dried roots of *Scutellariae baicalensis* Georgi. Glycyrrhizae Radix et Rhizoma, produced from the dried roots of plants of the family Leguminosae like *Glycyrrhiza uralensis* Fisch. Panax notoginseng Radix et Rhizoma (Sanqi) refers to the dried roots and rhizomes of *Panax notoginseng* (Burk.) F. H. Chen. All the Chinese medicinal materials were purchased Anhui Jiancheng Chinese Herbal Decoction Pieces Co., Ltd.

### Establishment of the T2DM rat model

After 1 week of adaptive feeding with a basal diet, 10 SD rats were selected as the normal control group (CK), and the remaining 50 rats were assigned to the modeling group. The normal control group (CK) continued to be fed with the basal diet, while the modeling group was fed a high-sugar and high-fat diet for 4 weeks to induce insulin resistance. After 4 weeks, the modeling group was fasted for 12 h with water allowed and then injected intraperitoneally with 25 mg/kg streptozocin (STZ) solution for two consecutive days based on their fasting body weight. On the third and sixth days after the injection, blood was collected from the tail vein to measure fasting blood glucose (FBG). Rats with FBG levels exceeding 11.0 mmol/L on both measurements were considered to have been successfully established as T2DM models. The study was approved by the Experimental Animal Ethics Committee of Guizhou Normal University (approval number: 2025030014).

### Animal grouping and drug administration protocols

Successfully modeled rats were randomly divided into five groups (*n* = 6 per group): the diabetic model group (MG), the metformin treatment group (MET), the fermented Coptis-notoginseng group (FRP), the fermented Coptis-Scutellaria group (FRS), and the fermented Coptis-Glycyrrhiza group (FRG). The CK group was fed a basal diet, while all other groups continued on the high-sugar, high-fat diet. The fermented Coptis herb pair products were ground into fine powder and suspended in physiological saline. The suspensions were administered by oral gavage once daily at a dose of 4.72 g/kg. The MET group received metformin at 120 mg/kg, and both the CK and MG groups were given an equal volume of physiological saline by gavage. All groups were administered continuous drug intervention for 6 weeks under routine feeding conditions and a 12 h light/dark cycle. The rats’ mental state, activity level, coat glossiness, and food intake were monitored daily, and fasting body weight was recorded weekly.

### Determination of fasting blood glucose (FBG) and oral glucose tolerance test (OGTT)

During the experiment, after drug administration, rats from all groups were fasted for 12 h with free access to water on days 0, 7, 14, 21, 28, 35, and 42. Blood samples were collected from the tail tip to measure FBG levels. On day 42 after continuous administration, following a 12 h fast with water available, the rats were orally administered a 50% glucose solution by gavage at a dose of 2.5 g per 100 g body weight. Blood glucose levels were measured and recorded immediately before (0 min) and at 30, 60, 90, and 120 min after glucose administration.

### Measurement of blood lipid levels

Following a 12 h fast with free access to water, all rats were anesthetized by intraperitoneal injection of 10% chloral hydrate, strictly accordance with animal ethics requirements. Upon successful anesthesia, the thoracic cavity was exposed by cutting the ribs. The pancreas and colonic contents were collected. Blood samples (6 mL per rat) were collected from the femoral artery, left to stand at room temperature for 1 h, and then centrifuged at 4000 rpm for 15 min to obtain serum. The separated serum was stored at −80 °C for subsequent determination of total cholesterol (TC), triglycerides (TG), high-density lipoprotein cholesterol (HDL-C), and low-density lipoprotein cholesterol (LDL-C).

### Histopathological observation of pancreatic tissue in T2DM rats

Pancreatic tissues were fixed in 4% paraformaldehyde solution, dehydrated, embedded in paraffin, and sectioned into 5 μm thin slices. Following deparaffinization and rehydration, tissue sections were stained with hematoxylin for 3 ~ 5 min, rinsed with tap water, differentiated in hydrochloric acid ethanol for 30 s, and immersed in tap water for 15 min. Subsequently, the sections were counterstained with eosin solution for 2 min, followed by dehydration, transparency, and mounting. The morphological structure and integrity of the pancreatic cells were examined under a light microscope. Pancreatic tissue injury was assessed using a scoring system ranging from 0 to 3, where 0 = normal islets, 1 = mild damage, 2 = moderate damage, and 3 = severe damage with fibrosis. Three replicates were included in each group, and statistical analysis was performed using non-parametric tests.

### Analysis of gut microbiota composition in T2DM rats

Genomic DNA of colonic contents collected from the rats was extracted using the E. Z. N. A.^®^ Soil DNA Kit (Omega Bio-tek, Norcross, GA, United States). The hypervariable region V3–V4 of the bacterial 16S rRNA gene were amplified with primer pairs 338F (5’-ACTCCTACGGGAGGCAGCAG-3′) and 806R (5’-GGACTACH VGGGTWTCTAAT-3′). Sequencing libraries were generated using the TruSeq^®^ DNA Sample Prep Kit, and the sequencing was performed on an Illumina Nextseq2000 platform (Illumina, San Diego, United States) to generate raw data. After demultiplexing, the resulting sequences were quality filtered with fastp (0.19.6) and merged with FLASH (v1.2.11). Then the high-quality sequences were de-noised using DADA2 plugin in the Qiime2 (version 2024) pipeline with recommended parameters. DADA2 denoised sequences are usually called amplicon sequence variants (ASVs). Taxonomic assignment of ASVs was performed using the Naive bayes consensus taxonomy classifier implemented in Qiime2 and the SILVA 16S rRNA database (v138.2).

Bioinformatic analysis of the gut microbiota was carried out using the Majorbio Cloud platform^1^. Alpha diversity indices and Good’s coverage were calculated with Mothur v1.30.1. Beta diversity was examined through principal coordinate analysis (PCoA) to elucidate variations in species composition among different sample groups. Venn diagrams were employed to illustrate shared and unique species compositions across experimental groups. The linear discriminant analysis (LDA) effect size (LEfSe)^2^ was performed to identify the significantly abundant taxa (phylum to genera) of bacteria among the different groups (LDA score>2, *p* < 0.05). Differences in species between the two groups were identified using inter-group difference tests (R-3.3.1).

### Statistical analysis

The data were analyzed using the SPSS Statistics 26.0 program (SPSS Inc., Chicago, IL, United States) through one-way analysis of variance (ANOVA) and the significance of differences between groups was analyzed using Tukey’s *post hoc* test. The data are expressed as the mean ± standard deviation (SD). Highly significant differences are indicated by *p* < 0.01, significant differences by *p* < 0.05.

## Results

### General status of rats in each group

Throughout the experimental period, the general status of the rats varied significantly among the groups. Rats in CK exhibited excellent mental state, normal activity, clean and white fur, well-formed feces, and normal urination. By contrast, rats in MG showed extremely poor mental state, characterized by lethargy, yellowish and severely shed fur. The bedding was excessively humid due to polyuria, and diarrhea emerged from the second week, resulting in a sour odor in the cage. Rats in MET displayed a relatively poor mental state, with withered, yellowish, and coarse fur, as well as a thin and weak physique. Although urine output improved compared to the MG, their feces remained poorly formed. Notably, the differences were observed among the various fermented Coptis herb pair groups. Rats in the FRP exhibited a relatively poor mental state, coarse and dull-yellow fur, and polyuria compared to the CK. Rats in the FRS also exhibited a relatively poor mental state and withered, yellowish fur with signs of hair loss. Their urine output was observed to be higher than CK, but diarrheal symptoms gradually improved by the fifth week. Rats in the FRG displayed a relatively better mental state, increased activity, and improved fur condition. These results suggest that fermented Coptis herb pairs may be superior to metformin in improving physical health of diabetic rats, with the fermented Coptis-Glycyrrhiza pair potentially offering the greatest benefit in ameliorating general health status.

### Effects of fermented Coptis herb pairs on body weight in T2DM rats

**Figure 1:**
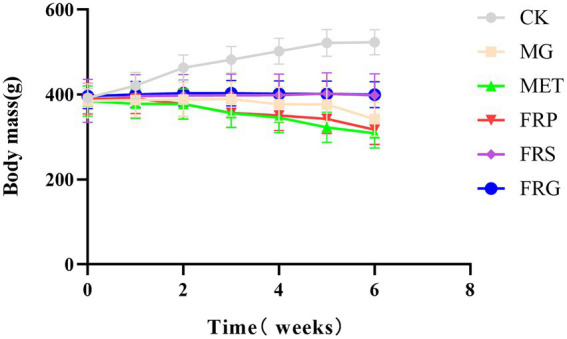
Effects on body weight of T2DM rats in each group. The gray, yellow, green, red, purple, and blue lines represent the normal, model, MET treated, fermented *Coptis-Notoginseng* treated, fermented *Coptis-Scutellaria* treated and fermented *Coptis-Glycyrrhiza* treated diabetic rats, respectively. Data are presented as the mean ± SD (*n* = 6).

During the experimental period, body weight of rats in the CK showed a gradual upward trend and was consistently higher than other T2DM groups. The body weights of rats in MG, FRP and MET exhibited a declining trend during the administration period. In contrast, the body weights of rats in the FRS and the FRG increased steadily. From the 4th week onward, the body weight of the MET was lower than all other groups, showing a significant difference compared to the FRS and the FRG (*p* < 0.05), and a highly significant decrease relative to the CK (*p* < 0.001). At the 4th, 5th, and 6th weeks, body weights of rats in the FRS and the FRG were significantly different from those in the MG, MET, and FRP (*p* < 0.05), whereas no significant difference was observed between the FRS and the FRG (*p* > 0.05) (Figure 1). These results indicate that both the fermented Coptis-Scutellaria and fermented Coptis-Glycyrrhiza herb pairs significantly ameliorate weight loss in T2DM rats, and their efficacy is superior to that of metformin and the fermented Coptis-Notoginseng herb pair.

### Effects of fermented Coptis herb pairs on fasting blood glucose (FBG) in T2DM rats

**Figure 2:**
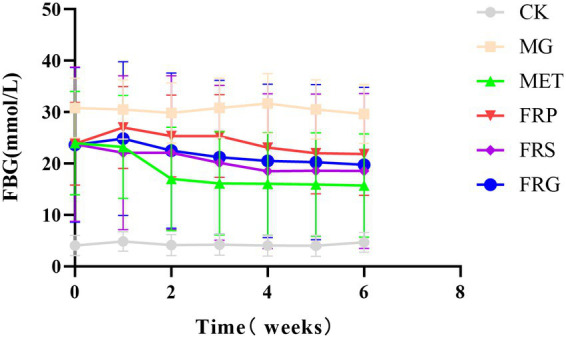
The fasting blood glucose (FBG) levels in rats of each group. Data are presented as the mean ± SD (*n* = 6).

The Fasting Blood Glucose (FBG) levels of successfully modeled T2DM rats were significantly higher than those in the CK (*p* < 0.001). The MG maintained high blood glucose levels, approximately 23.8 mmol/L, with minimal fluctuation. During the first week of administration, interventions in all drug groups had little effect on FBG values. In the FRP, FBG levels showed a transient increase, followed by a decrease only after the fourth week. From the third to the sixth week, FBG levels in both the FRS and FRG exhibited a downward trend, with no statistically significant difference between the two groups. Both the FRS and FRG exhibited significantly lower FBG levels than the FRP. Meanwhile, both these two groups showed higher FBG levels than the MET, although the difference was not statistically significant. During the fourth and fifth weeks, FBG levels of rats in FRS were significantly lower than MG (*p* < 0.05) (Figure 2).

These results indicate that both the fermented Coptis-Scutellaria and fermented Coptis-Glycyrrhiza pairs exerted notable regulatory effects on the FBG levels in T2DM rats. Among these, the fermented Coptis-Scutellaria pair exhibited the strongest efficacy, followed by the Coptis-Glycyrrhiza pair, both of which outperformed the fermented Coptis-Notoginseng pair. However, in terms of hypoglycemic efficacy alone, each of the three fermented herb pairs was less effective than metformin.

### Effects of fermented Coptis herb pairs on oral glucose tolerance test (OGTT) in T2DM rats

**Figure 3:**
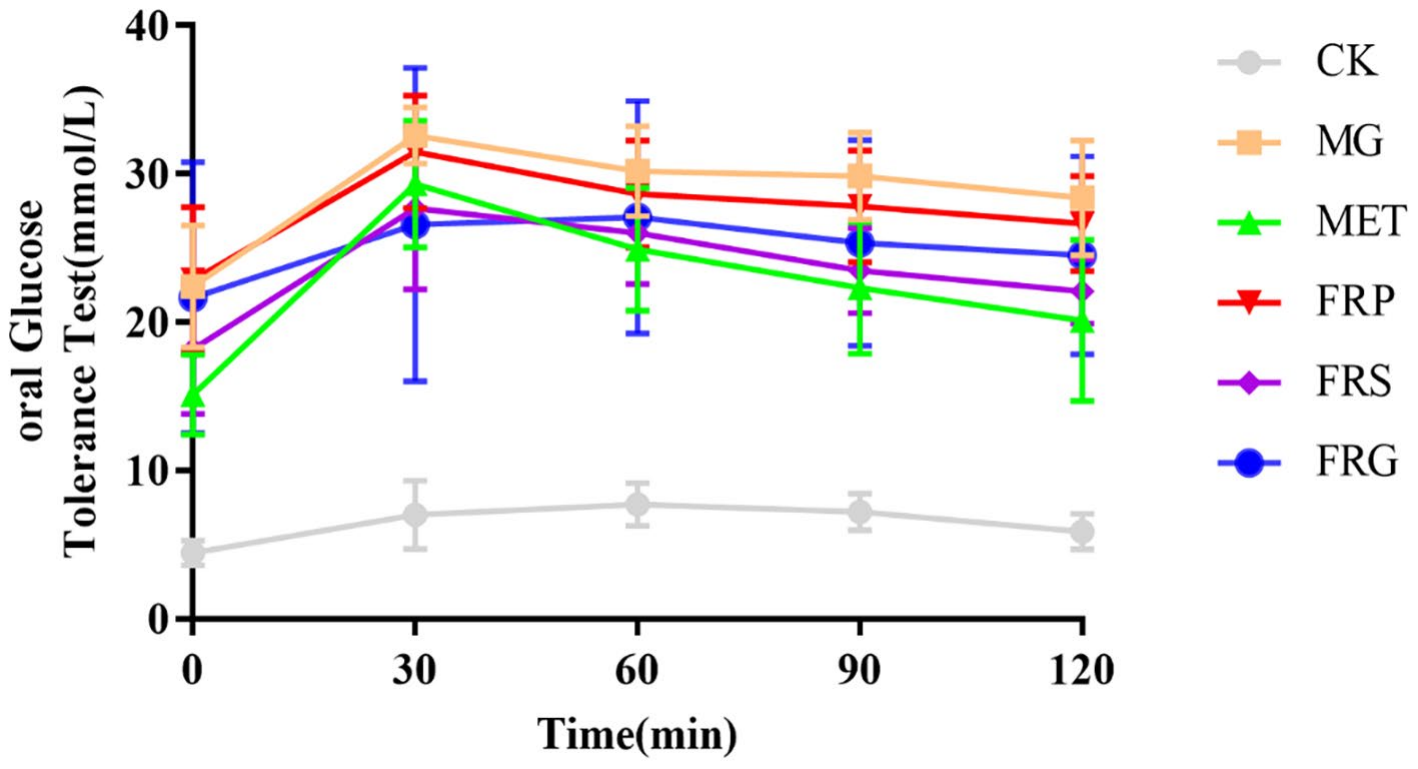
Blood glucose levels of T2DM rats in the oral glucose tolerance test (OGTT). Data are presented as mean ± SD and were analyzed using one-way ANOVA followed by Tukey’s *post hoc* test.

Both metformin and the three fermented Coptis herb pairs reduced the OGTT values in T2DM rats. Blood glucose level in MET increased more sharply within 30 min than the other five groups, and its glucose value at the 30 min time point was higher than in the FRS and FRG groups. After 30 min, however, blood glucose in the MET declined more rapidly than in the other groups. In the FRP, FRS, and FRG, blood glucose peaked at 30 min and subsequently decreased. At the 30 min time point, the FRP showed the highest blood glucose value and the slowest decline thereafter (Figure 3). In contrast, the FRG exhibited a gradual rise in blood glucose, the lowest value at 30 min, and a gentle decrease afterwards. The fermented Coptis-Scutellaria and Coptis-Glycyrrhiza herb pairs exhibited superior efficacy in the Oral Glucose Tolerance Test (OGTT) compared to the fermented Coptis-Notoginseng pair.

### Effects of fermented Coptis herb pairs on blood lipid levels in T2DM rats

**Figure 4:**
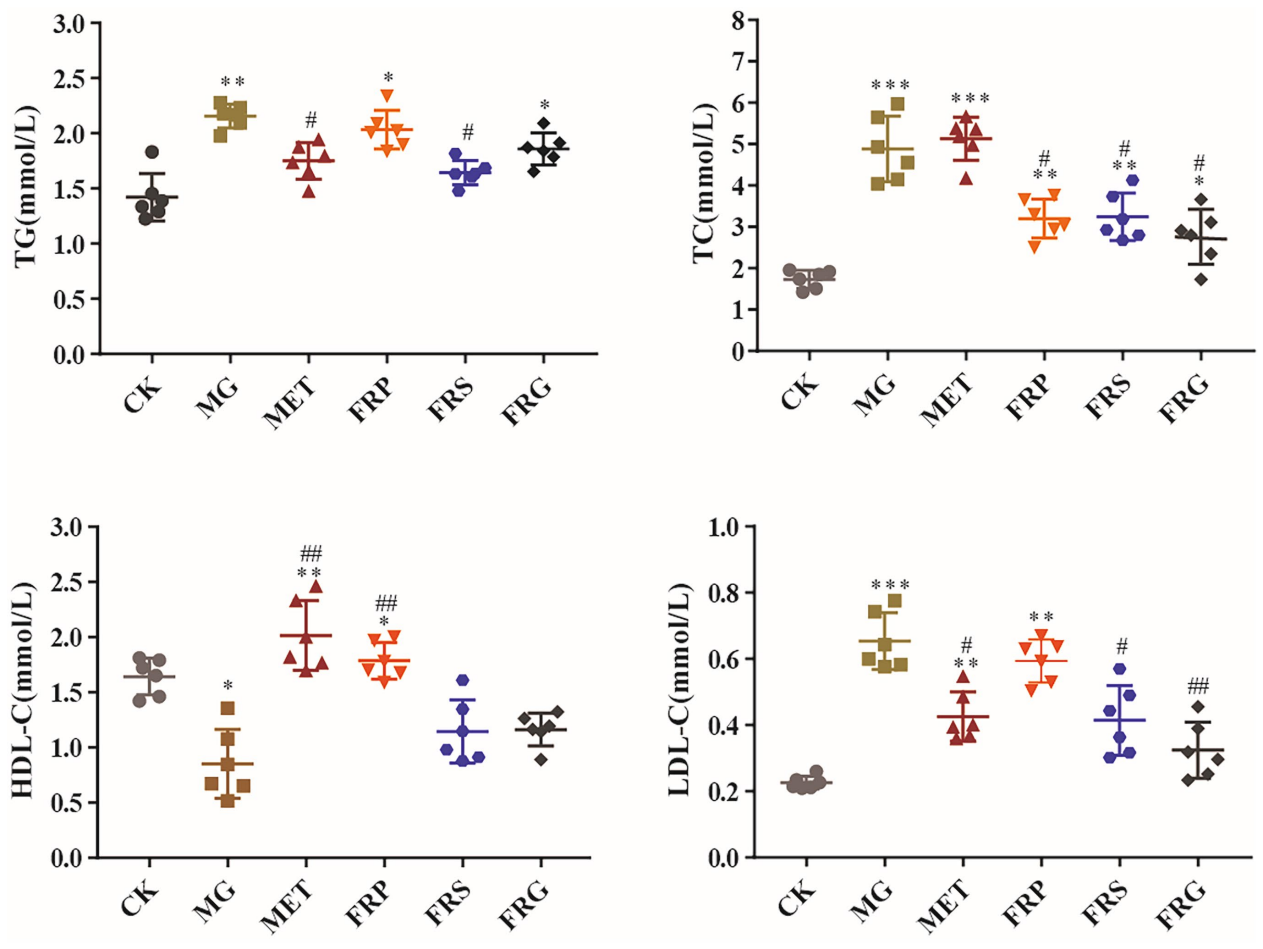
Blood lipid levels of rats in each group. TG, triacylglycerol; TC, total cholesterol; HDL-C, high-density lipoprotein cholesterol; LDL-C, low-density lipoprotein cholesterol. Data were analyzed using one-way ANOVA followed by the Tukey’s post hoc test. ^*^p < 0.05, ^**^p < 0.01, ^***^p < 0.001 vs. CK; ^#^*p* < 0.05, ^##^*p* < 0.01, ^###^*p* < 0.001 vs. MG.

Compared with the CK, Triglyceride (TG) levels were significantly increased in the MG, FRP and FRG (*p* < 0.01). Compared with the MG, TG levels in MET and FRS were significantly decreased (*p* < 0.01), while no significant differences were observed (*p* > 0.05) between the FRP and FRG. These results indicated that metformin and the fermented Coptis-Scutellaria herb pair significantly improved TG levels in T2DM rats. Compared with the CK, total cholesterol (TC) levels in the MG, FRP, FRS, and FRG were significantly increased. Compared with the MG, TC levels in the FRP, FRS, and FRG, were significantly decreased (*p* < 0.05), while no significant change was observed (*p* > 0.05) in the MET. These results demonstrated that all three fermented Coptis herb pairs efficiently regulated TC in T2DM rats, with effects superior to those of metformin, although their effects varied (Figure 4).

High density lipoprotein cholesterol (HDL-C), often referred to as “good cholesterol,” is involved in lipid metabolism, facilitates the conversion of cholesterol into bile acids in the liver or its direct excretion via bile from the intestines. As an anti-atherogenic plasma lipoprotein, a higher HDL-C level indicates better blood lipid metabolism. Compared with the CK, the HDL-C level was significantly decreased (*p* < 0.01) in the MG. Compared with the MG, HDL-C levels were significantly increased (*p* < 0.01) in the MET and FRP, indicating a positive effect on HDL-C regulation in T2DM rats. In contrast, no significant increase was observed in the FRS and FRG, suggesting that these two herb pairs had limited regulatory effects on HDL-C in T2DM rats.

Compared with the CK, low-density lipoprotein cholesterol (LDL-C) levels were significantly increased (*p* < 0.01) in the MG and FRP. Compared with the MG, LDL-C levels were significantly decreased (*p* < 0.05) in the MET, FRS and FRG. These findings indicate that metformin, fermented Coptis-Scutellaria, and fermented Coptis-Glycyrrhiza herb pairs significantly ameliorated LDL-C metabolism in T2DM to varying degrees, with the fermented Coptis-Glycyrrhiza herb pair demonstrating the most pronounced efficacy.

### Repair effects of fermented Coptis herb pair on pancreatic tissue in T2DM rats

**Figure 5:**
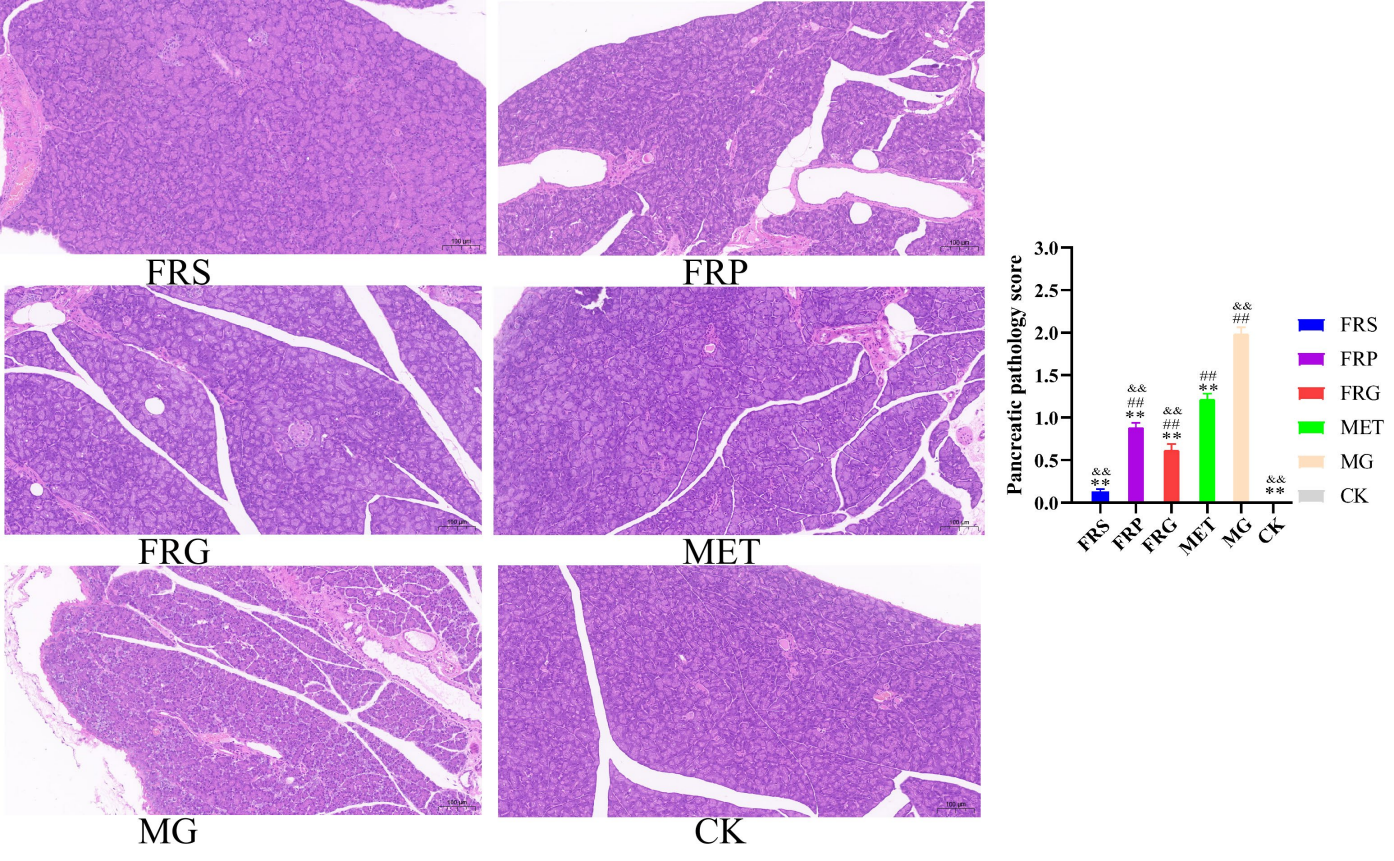
Characteristic of pancreatic tissues from rats in each group (100× magnification). Hematoxylin–eosin (H&E) staining of the pancreas, scale bar = 100 μm (*n* = 3). ^*^p < 0.05, ^**^p < 0.01 vs. MG; ^#^*p* < 0.05, ^##^*p* < 0.01 vs. CK, ^&^*p* < 0.05, ^&&^*p* < 0.01 vs. MET.

Pancreatic injury was assessed and scored based on the morphological characteristics of the pancreatic tissue and islet cells (Figure 5). In the CK, pancreatic tissues exhibited an intact and regular cellular architecture, the islet cells appeared round or oval with clearly defined borders and centrally located nuclei, and showed no edema, abundant acinar cells were closely arranged. In the MG, the islets showed a significant reduction in cell number, disordered arrangement with inflammatory infiltration, visible degeneration and necrosis, atrophy with indistinct borders, along with widened intercellular spaces and edematous, atrophic acinar cells. In the FRS, islet cells were relatively regularly arranged with distinct nuclei, occasionally showing inflammatory cell infiltration and cytoplasmic vacuolar degeneration. In the FRP, islet cells were irregularly arranged, with minor necrosis accompanied by inflammatory cell infiltration. In the FRG, the pancreatic tissue exhibited relatively dense and uniform, with occasional vacuolar degeneration observed in the islet cells. In the MET, the arrangement of islet cells was relatively irregular, but overall organized, with occasional observations of degeneration, necrosis, and vacuolization. All fermented Coptis herb pairs ameliorated islet cell degeneration, necrosis, and inflammatory cell infiltration in T2DM rats.

### Effects of fermented Coptis herb pairs on the gut microbiota in T2DM rats

#### Abundance and diversity of the gut microbiota

A total of 1,815,196 clean reads were obtained from 16S rRNA amplicon sequencing of colonic contents from a total of 36 samples, with an average of 413 ~ 428 reads per sample. The alpha diversity of the microbial communities in the samples was represented by the Chao1, ACE indices, Shannon, and Simpson indices. The α-diversity index is summarized in Table 1, and a Good’s coverage of 0.999 indicated that most microorganisms were present in the samples. The Chao1 and ACE indices of the FRS were significantly higher than those of the MG, indicating increased species richness and a greater number of species in the gut microbiota of the FRS. Although the Chao1 and ACE indices of the FRG and FRP were also higher than those of the MG, the differences were not significant. A higher Shannon index indicates greater diversity of the microbial community in the samples, while the Simpson index exhibits the opposite trend. The Shannon index of the FRS was significantly higher, and the Simpson index significantly lower, than those of the CK, suggesting high species diversity and even distribution in the FRS. The FRG and FRP exhibited higher Shannon indices and lower Simpson indices compared to the CK, although the differences were not significant.

**Table 1:** Effects of fermented Coptis herb pairs on alpha diversity of gut microbiota in T2MD rats.

Index	ACE	Chao1	Sobs	Shannon	Simpson
CK	311.99 ± 171.02	311.50 ± 170.84	311.17 ± 170.30	3.16 ± 1.25	0.15 ± 0.11^&^
FRG	330.62 ± 80.54	330.33 ± 80.49	328.50 ± 80.83	3.81 ± 0.42	0.09 ± 0.05^&^
FRP	366.18 ± 55.96	365.71 ± 55.87	364.50 ± 56.65	4.14 ± 0.44	0.05 ± 0.03
FRS	398.43 ± 58.66^#^	397.67 ± 58.67^#^	396.33 ± 57.96^#^	4.51 ± 0.30^* ##^	0.03 ± 0.01^*#^
MET	331.29 ± 46.49	330.73 ± 46.62	329.17 ± 46.40	4.24 ± 0.26^#^	0.04 ± 0.01^#^
MG	288.20 ± 67.60	287.37 ± 67.51	286.33 ± 67.03	3.63 ± 0.56	0.09 ± 0.07

^*^p < 0.05 vs. CK group; ^#^*p* < 0.05 vs. MG group; ^##^*p* < 0.01 vs. MG group; ^&^*p* < 0.05 vs. MET group. Data are presented as Mean ± SD (*n* = 6).

**Figure 6:**
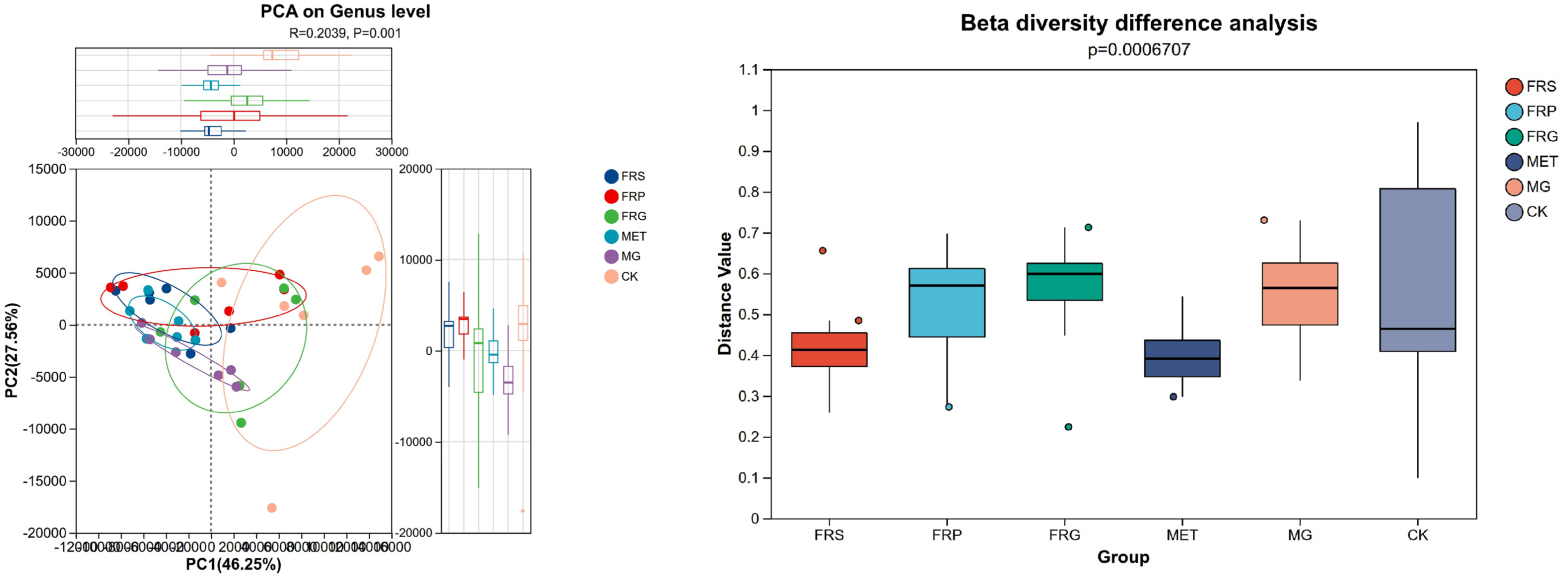
PCoA plot of β-diversity of the gut microbiome in T2DM rats. *R* > 0 indicates that the between group differences were greater than within-group differences. Statistical significance was assessed by PERMANOVA (*p*-value).

Principal coordinate analysis (PCoA) revealed distinct clustering among the groups, indicating significant differences in microbial community composition between the CK and the FRG, FRP, FRS, MET, MG. Notably, the microbial communities of the FRG and FRP clustered more closely with the CK, suggesting a stronger regulatory effect on dysbiotic gut microbiota (Figure 6).

### Effects of fermented Coptis herb pairs on the microbial communities composition in T2DM rats

**Figure 7:**
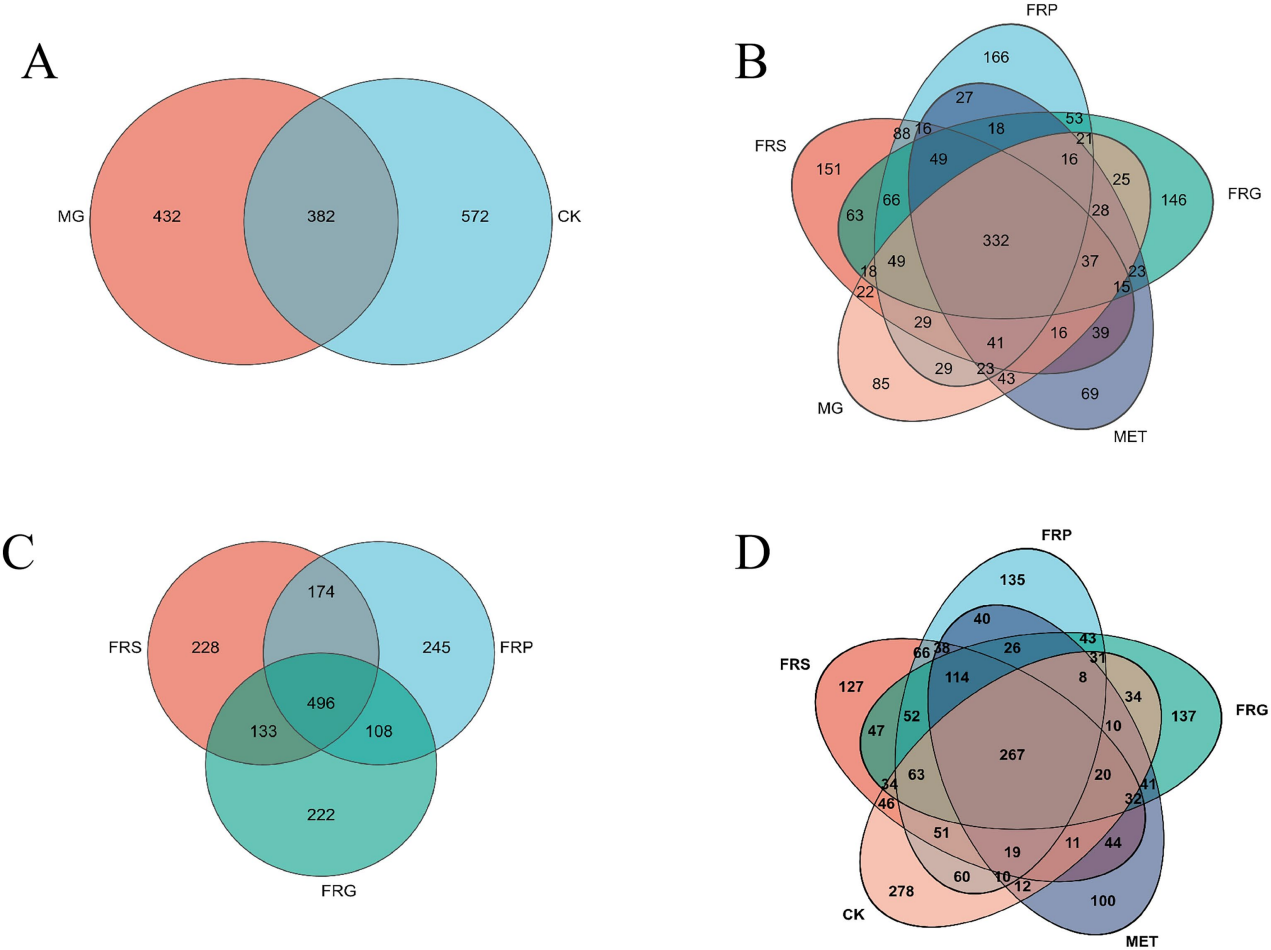
Venn diagram of amplicon sequence variants (ASVs) of gut microbiota in rats from each group. **(A)** Venn diagram showing shared and unique ASVs between the MG and CK groups. **(B)** Among the MG, FRS, FRP, FRG, and MET groups. **(C)** Among the FRS, FRP, and FRG groups. **(D)** Among the CK, FRS, FRP, FRG, and MET groups.

A comparative analysis of shared and unique amplicon sequence variant (ASV) among groups was conducted (Figure 7). Compared to the CK, the MG lost 140 ASVs and gained 432 ASVs. The three fermented Coptis herb pair groups (FRS, FRP, and FRG) shared 496 common ASVs and possessed 228, 245, and 222 unique ASVs, respectively, with the FRP group exhibiting the highest number of unique ASVs (245). Overall, treatment with metformin or fermented Coptis herb pairs increased the number of unique gut microbial taxa in T2DM rats to varying degrees, with the MET showing the most pronounced increase.

**Figure 8:**
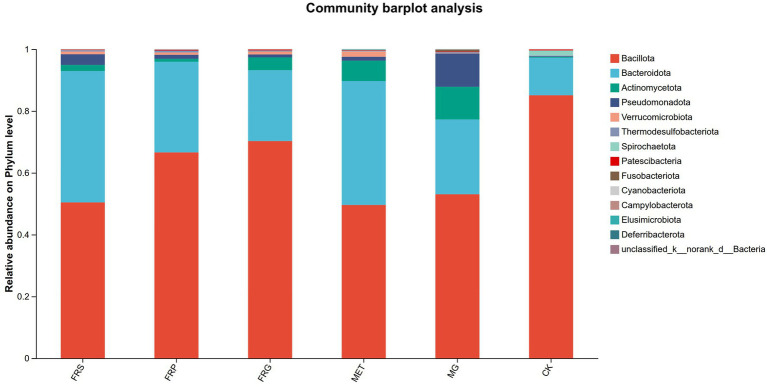
The relative abundances of microbial species in each group of rats at the phylum level. The *X*-axis represents group names, and the *Y*-axis represents the proportion of each species in the samples of the corresponding group. Different colors indicate different species, and the length of the bars represents the relative abundance of each species.

At the phylum level, the dominant phyla were Bacillota (49.58–70.26%), Bacteroidota (12.15–42.50%), and Actinomycetota (0.94–10.57%). Bacillota remained the most abundant phylum in the MET, FRS, FRP, and FRG groups, with relative abundances of 49.58, 50.42, 66.60, and 70.26%, respectively. Compared with the CK, the MG exhibited significantly higher abundances of potentially harmful phyla, including Pseudomonadota (10.74%) and Actinomycetota (10.57%). Treatment with metformin or fermented Coptis herb pairs (FRS, FRP, and FRG) significantly reduced the abundances of these pathogenic phyla, with the FRG group showing the most pronounced reduction (Figure 8).

**Figure 9:**
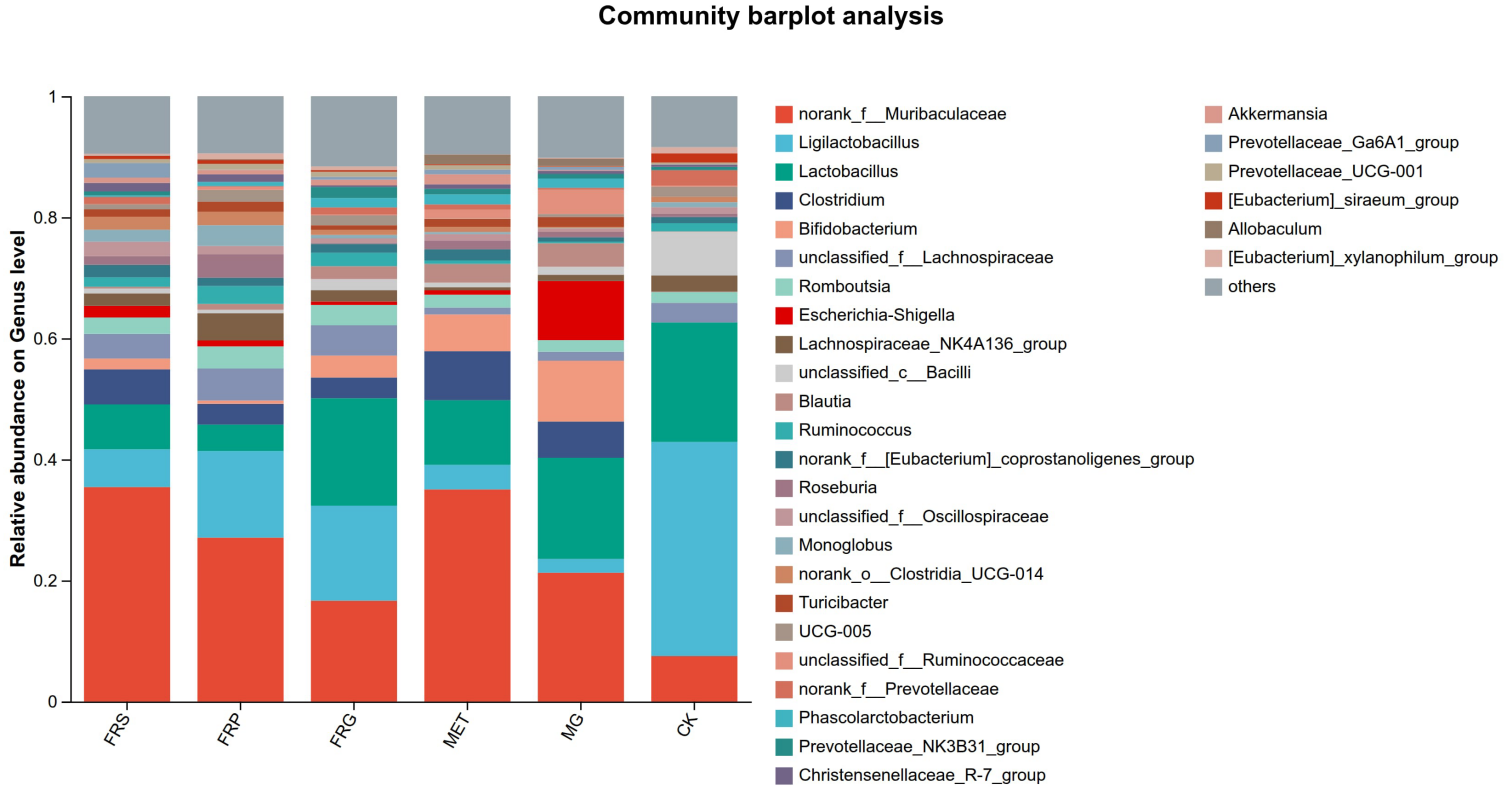
Relative abundances of gut microbial genera in each group at the genus level. Each color represents a specific genus, and the bar length indicates its relative abundance within the group.

**Figure 10:**
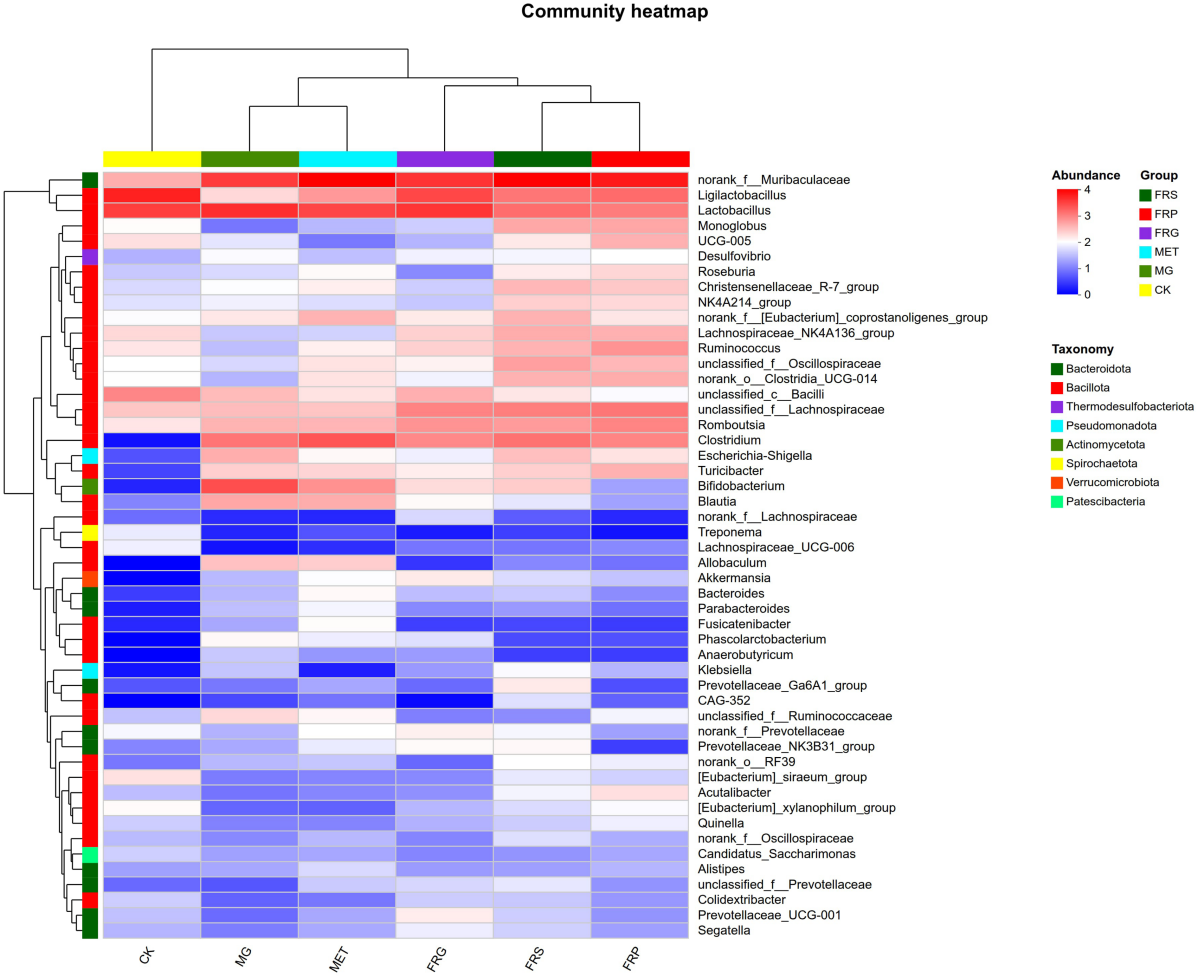
Heatmap showing the distribution of dominant microbial genera across groups. The color gradient represents the relative abundance of each genus, as indicated by the legend.

We further analyzed the differences of gut microbiota composition among groups at the genus level (Figure 9). Compared with the CK, the MG exhibited an increased abundance of several potentially harmful microbial genera, including norank_f_*Muribaculaceae*, *Clostridium*, *Bifidobacterium*, *Blautia*, *Escherichia-Shigella*, and *Allobaculum*, while the abundance of beneficial gut microbial genera such as *Ligilactobacillus*, unclassified_c_ *Bacilli*, *Lactobacillus*, *Lachnospiraceae*_NK4A136_group, unclassified_f_*Lachnospiraceae*, and *Monoglobus* were decreased (*p* < 0.05) (Figure 10). Compared with the MG, the fermented Coptis-Scutellaria, fermented Coptis-notoginseng, and fermented Coptis-Glycyrrhiza herb pairs reduced the abundance of harmful microbial genera such as *Bifidobacterium*, *Allobaculum*, unclassified_f_*Ruminococcaeae* and *Blautia*, while significantly increased the abundance of beneficialgenera including *Romboutsia*, *Ruminococcus*, unclassified_f_*Lachnospiraceae*, *Monoglobus* and unclassified_f_*Oscillospiraceae*. Compared with the MET, the fermented Coptis-Glycyrrhiza herb pairs showed a significant increase in the abundance of beneficial microbial genera such as *Ligilactobacillus, Akkermansia, Prevotellaceae*_UGG-001, whereas the fermented Coptis-notoginseng and fermented Coptis-Scutellaria groups exhibited a significant increase in the abundance of *Ruminococcus*, unclassified_f_*Oscillospiraceae*.

### Differences of the gut microbiota composition in T2DM rats

**Figure 11:**
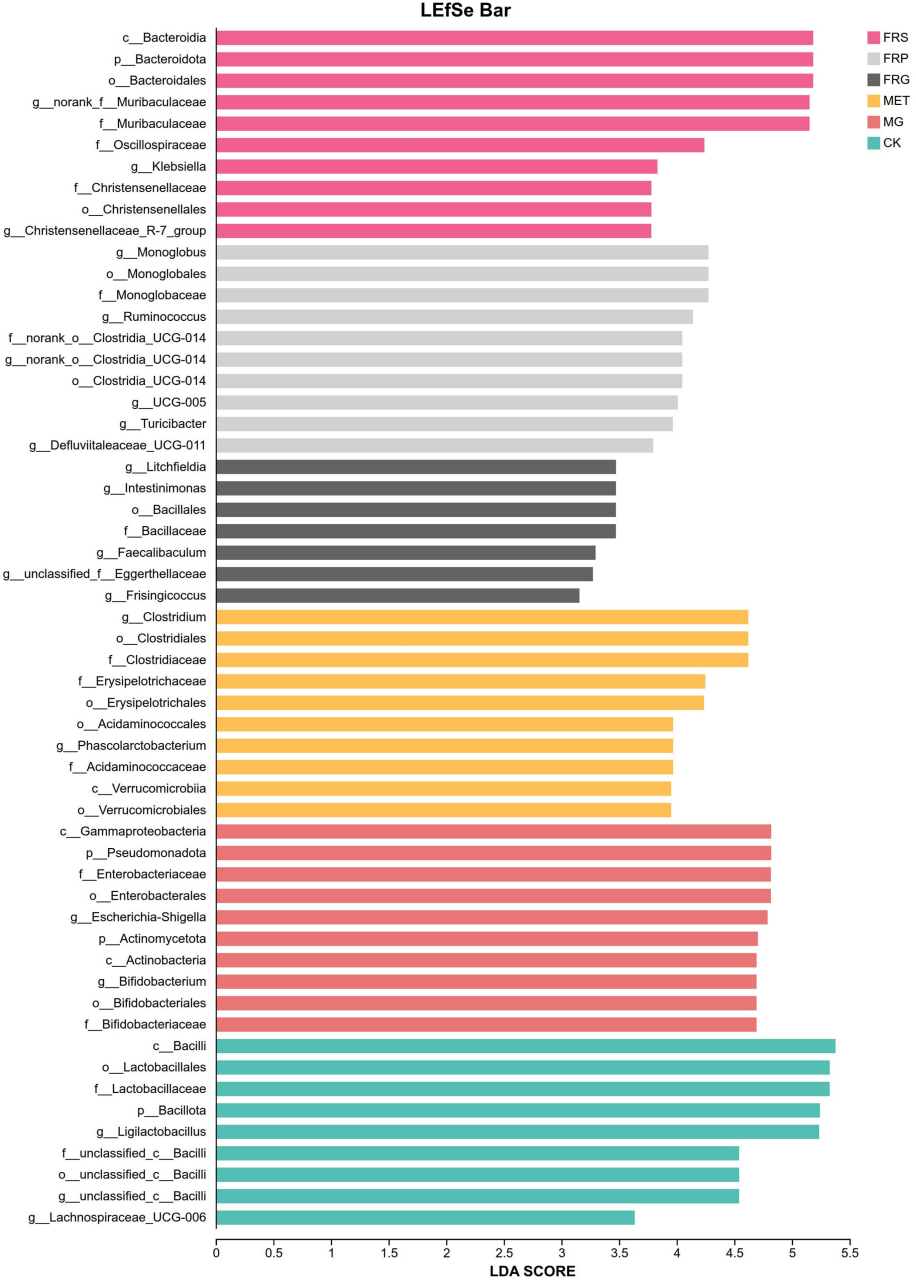
The LDA scores of the significantly different microbial species at the genus level across all groups.

The differences in the gut microbiota composition among T2DM rats treated with different fermented Coptis herb pairs were significant, characterized by a marked increase in beneficial genera and a notable decrease in harmful genera (Figure 11). Compared with MG, the FRS showed significantly increased abundance of unclassified_f_*Oscillospiraceae*, norank_o_*Clostridia*_UCG-014, *Monoglobus*, *Ruminococcus*, and unclassified_f_*Enterobacteriaceae*, while the abundance of potential opportunistic pathogens such as *Bifidobacterium*, unclassified_f_*Ruminococcaceae*, *Phascolarctobacterium*, and *Allobaculum* were significantly decreased (*p* < 0.05). Compared with the MG, the genera that significantly increased in the FRP included *Lachnospiraceae*_NK4A136_group, *Monoglobus*, *Ruminococcus*, norank_o_*Clostridia*_UCG-014, *Acutalibacter*, etc., whereas the genera that significantly decreased included *Lactobacillus*, *Bifidobacterium*, *Phascolarctobacterium*, unclassified_c_*Bacilli*, *Allobaculum*, etc. (*p* < 0.05). Compared with the MG, the FRG exhibited a significantly different microbial profile, characterized by a higher abundance of *Ligilactobacillus*, norank_f_*Lachnospiraceae*, *Akkermansia*, and *Prevotellaceae*_UCG-001, and a lower abundance of unclassified_f_*Ruminococcaceae*, *Allobaculum*, and *Extibacter* (*p* < 0.05).

**Figure 12:**
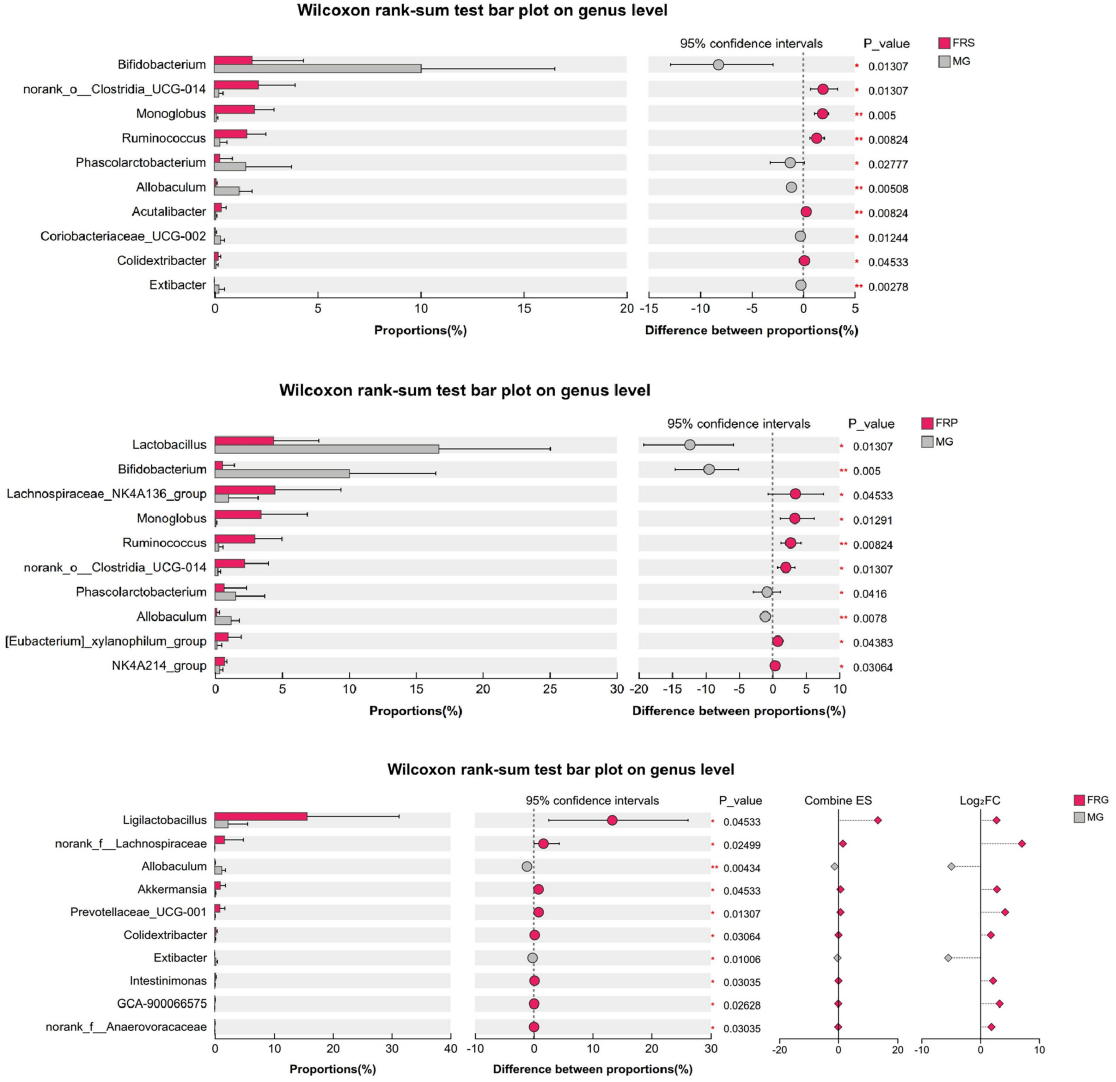
Differences in microbial species between groups at the genus level: **(A)** comparison between the FRS and MG; **(B)** comparison between the FRP and MG; **(C)** comparison between the FRG and MG.

To further elucidate the differential regulatory effects of fermented Coptidis herb pairs on the gut microbiota in T2DM rats, a pairwise comparison analysis was conducted between each fermented Coptis herb pair groups and the CK. Compared to the CK, the FRS exhibited a significant enrichment of norank_f_*Muribaculaceae*, *Clostridium*, *Prevotellaceae*_Ga6A1_group, *Escherichia*-*Shigella*, *Bifidobacterium*, *Akkermansia*, and *Christensenellaceae*_R-7_group, alongside a significant decrease of *Ligilactobacillus* and unclassified_c_*Bacilli.* In the FRP, the genera with higher abundance relative to CK included norank_f_*Muribaculaceae*, *Clostridium*, *Turicibacter*, *Acutalibacter*, and *Akkermansia*, while unclassified_c_*Bacilli* and *Treponema* were present in lower abundance. For the FRG, a higher abundance was observed for *Bifidobacterium*, *Clostridium*, *Phascolarctobacterium*, *Akkermansia*, *Turicibacter*, *Megamonas*, and *Faecalibaculum*, whereas the [*Eubacterium*]_*siraeum*_group and *Treponema* were significantly decreased (Figure 12). In summary, the fermentation of different Coptis herb pairs with *E. cristatum* positively modulated the disordered gut microbiota in T2DM rats by promoting the proliferation of various beneficial microbial species. However, the different fermented Coptis herb pairs led to distinct gut microbiota profiles, which might explain the differences in their hypoglycemic activities.

## Discussion

Type 2 diabetes mellitus (T2DM) is a metabolic disease with complex pathogenesis, and has become a major serious threat to public health. Patients in the middle and late stages exhibit severe gut microbiota dysbiosis, characterized by an increase of harmful microbial species and significant decrease in the diversity and abundance of probiotic microbial species, also accompanied by disordered glucose and lipid metabolism, impaired pancreatic cells, and systemic low grade inflammation. Therefore, effective therapeutic strategies for T2DM include controlling blood glucose levels, correcting dyslipidemia, repairing pancreatic tissue, and regulating the gut microbiota. Coptidis Rhizoma is a representative herb in TCM for treating T2DM, usually used in combination with other Chinese herbs, though its hypoglycemic activity remains limited. Fermentation of Coptidis Rhizoma with probiotics is an effective strategy to enhance its hypoglycemic activity, but screening for suitable strains is particularly challenging due to the strong antimicrobial activity of berberine. Our results indicated that *E. cristatum* thrives on various Coptidis Rhizoma pair substrates. Particularly, the FRG and FRS demonstrate significant hypoglycemic activity and pancreatic protective effects, confirming that *E. cristatum* is one of the most excellent probiotic strains for fermenting Coptidis-based formulations. Currently, Metformin remains the first-line clinical agent for treating T2DM, but it often causes significant gastrointestinal side effects and excessive weight loss in patients, which can lead to compromised immunity. Maintaining a certain body weight is particularly important for elderly patients with severe diabetes. In this regard, FRS and FRG demonstrated superior performance over both metformin and FRP in terms of weight maintenance. Although metformin exhibits strong blood glucose control, the fermented Coptis herb pairs show benefits in overall physical conditions such as mental state and coat glossiness, especially the FRG and FRS. Recent treatment concepts for diabetes have shifted from focusing solely on glycemic control to emphasizing systemic organ protection, pancreatic repair, and gut microbiota regulation.

Clinically, the hypoglycemic efficacy of traditional Chinese medicinal herbs such as Coptidis Rhizoma, Scutellariae Radix, and Glycyrrhizae Radix remains quite limited, often requiring high doses to achieve effective blood glucose control. In this study, both the fermented Coptidis-Scutellariae and the fermented Coptidis-Glycyrrhizae demonstrated strong hypoglycemic effects at conventional doses. Based on OGTT levels, the MET showed pronounced fluctuation in blood glucose rise and fall, while the three fermented Coptis herb pairs exhibited smaller fluctuations in blood glucose changes compared to MET, demonstrating advantages in glycemic stability.

T2DM ultimately leads to severe disorders in lipid metabolism. Our finding indicated that different Coptis herb pairs exhibit varying capacities in regulating these disorders. FRS, FRG, and FRP showed significant regulatory effects on total cholesterol (TC) in T2DM rats, outperforming metformin. The elevated TC levels in the MG rats are likely due to hyperglycemia-induced liver injury, which disrupts TC metabolism. All three fermented Coptis herb pairs demonstrated a certain degree of ability to repair liver damage. Triglycerides (TG), the transesterification products of fatty acids and glycerol, play a crucial role in lipid transport. The elevated TG levels observed in T2DM rats may be attributed to the accumulation of fatty acid ester metabolites, which impair β-cell function. Excessive TG accumulation in pancreatic islets interferes with glucose metabolism, reduces insulin secretion, and consequently affects the regulation of triglycerides in rats (Ye et al., 2019). The fermented Coptis-Scutellaria likely repaired some pancreatic β-cells, leading to a significant improvement in TG levels in T2DM rats. Panax notoginseng Radix et Rhizoma (Sanqi) is a traditional Chinese medicine used to regulate lipid metabolism, without particularly outstanding hypoglycemic activity and rarely used in hypoglycemic prescriptions. In this study, the fermented Coptis-notoginseng exerts better hypoglycemic activity and strong regulatory effect on high-density lipoprotein, making it suitable for treating diabetes accompanied by abnormal high-density lipoprotein.

Gut microbiota homeostasis plays a crucial role in regulating glucose and lipid metabolism. Traditional Chinese herbs, probiotic, and various active components can reverse the progression of T2DM by modulating the gut microbiota, influencing short-chain fatty acid (SCFA) production, improving gut microbiota metabolites, and ameliorating the disorder of glucose and lipid metabolism (Clementino et al., 2025). Different fermented Coptidis herb-pairs exert distinct effects on the gut microbiota, with significant differences observed in their composition. This variation accounts for their differential abilities to ameliorate glucose and lipid metabolism disorders. The fermented Coptis-Scutellaria significantly enriched beneficial bacterial genera, specifically unclassified_f_*Oscillospiraceae*, norank_o_*Clostridia*_UCG-014, *Monoglobus*, and *Ruminococcus*, indicating that the gut ecosystem possesses an enhanced capacity for dietary fiber fermentation and SCFA production. In the FRP, the beneficial bacterial genera that significantly increased were *Lachnospiraceae*_NK4A136_group, *Monoglobus*, *Ruminococcus*, norank_o_*Clostridia*_UCG-014, and *Acutalibacter.* The *Lachnospiraceae*_NK4A136_group is an important butyrate producer that strengthens the intestinal barrier, reduces “leaky gut,” and participates in the regulation of glucose and lipid metabolism through the gut microbiota-SCFA-GPR41-GLP-1 axis (Zhang et al., 2024). The FRG exhibited a significantly distinct beneficial microbial profile, characterized by a higher abundance of *Ligilactobacillus*, norank_f_*Lachnospiraceae*, *Akkermansia*, and *Prevotellaceae*_UCG-001. Among these, *Ligilactobacillus* was notable for its ability to rapidly repair damaged intestinal epithelial cells and strongly inhibit enteric pathogenic bacteria (Wang et al., 2025). The fermented Coptis-Glycyrrhiza exhibited better glycemic control and ameliorated weight loss, which suggests that fermented Coptis-Glycyrrhiza may exert its therapeutic effects by precisely regulating these beneficial microbial species.

Short-chain fatty acids, derived from gut microbiota fermentation of dietary fiber, are closely related to the host’s immune system and glucose-lipid metabolism. An increase in the abundance of SCFA-producing gut microbiota is a potential therapeutic approach for T2DM (Min et al., 2022). In the present study, the abundance of unclassified_f_*Oscillospiraceae* increased significantly in the FRS group, and the abundance of *Prevotellaceae* increased significantly in the FRG group, both of which are SCFA-producing bacteria. *Akkermansiaceae*, one of the most promising potential probiotics colonizing the mucosal layer, plays an important role in gastrointestinal homeostasis and metabolic balance by regulating intestinal permeability, improving glucose tolerance, and promoting insulin secretion (Liu et al., 2020). This study demonstrated that administration of fermented fermented Coptis-Glycyrrhiza to T2DM rats significantly increased the abundance of *Akkermansiaceae.* Therefore, increasing the abundance of intestinal *Akkermansiaceae* may be one of the mechanisms through which fermented Coptis herb pairs exert their therapeutic effects in T2DM rats.

## Conclusion

In conclusion, different fermented Coptis herb pairs distinctly shape the gut microbiota, leading to varied regulatory effects on glucose and lipid metabolism. Both fermented Coptis-Scutellaria and fermented Coptis-Glycyrrhiza outperformed metformin and fermented Coptis-notoginseng in regulating metabolic disorders. Among them, fermented Coptis-Glycyrrhiza was most effective at promoting beneficial microbes, while fermented Coptis-notoginseng showed superior lipid regulation, especially in increasing high-density lipoprotein (HDL). These findings provide a theoretical basis for using fermented Coptis formulations to manage blood glucose and offer new insights into TCM-based diabetes treatment.

## Data Availability

The raw 16S rRNA gene sequencing data generated in this study have been deposited in the NCBI Sequence Read Archive (SRA) under accession number PRJNA1377890 (https://www.ncbi.nlm.nih.gov/bioproject/PRJNA1377890). All other data generated or analyzed during the current study are included in the article and its supplementary materials.

## Footnotes

1
https://cloud.majorbio.com

2
http://huttenhower.sph.harvard.edu/LEfSe
